# Sapogenol is a Major Microbial Metabolite in Human Plasma Associated with High Protein Soy-Based Diets: The Relevance for Functional Food Formulations

**DOI:** 10.3390/foods9040422

**Published:** 2020-04-03

**Authors:** Madalina Neacsu, Vassilios Raikos, Yara Benavides-Paz, Sylvia H. Duncan, Gary J. Duncan, James S. Christie, Alexandra M. Johnstone, Wendy R. Russell

**Affiliations:** Rowett Institute, University of Aberdeen, Foresterhill, Aberdeen AB25 2ZD, Scotland, UK; v.raikos@abdn.ac.uk (V.R.); ylbenavides@gmail.com (Y.B.-P.); sylvia.duncan@abdn.ac.uk (S.H.D.); gary.duncan@abdn.ac.uk (G.J.D.); j.christie@abdn.ac.uk (J.S.C.); alex.johnstone@abdn.ac.uk (A.M.J.); w.russell@abdn.ac.uk (W.R.R.)

**Keywords:** soybean, saponin, sapogenol, gut metabolism, bioavailability, human study, functional food

## Abstract

Legumes are a source of health-promoting macro- and micronutrients, but also contain numerous phytochemicals with useful biological activities, an example of which are saponins. Epidemiological studies suggest that saponins may play a role in protection from cancer and benefit human health by lowering cholesterol. Therefore, they could represent good candidates for specialised functional foods. Following the consumption of a soya-rich high-protein weight-loss diet (SOYA HP WL), the concentrations of Soyasaponin I (SSI) and soyasapogenol B (SSB) were determined in faecal samples from human volunteers (n = 10) and found to be between 1.4 and 17.5 mg per 100 g fresh faecal sample. SSB was the major metabolite identified in volunteers’ plasma (n = 10) after consumption of the soya test meal (SOYA MEAL); the postprandial (3 h after meal) plasma concentration for SSB varied between 48.5 ng/mL to 103.2 ng/mL. The metabolism of SSI by the gut microbiota (in vitro) was also confirmed. This study shows that the main systemic metabolites of soyasaponin are absorbed from the gut and that they are bioavailable in plasma predominantly as conjugates of sapogenol. The metabolism and bioavailability of biologically active molecules represent key information necessary for the efficient development of functional foods.

## 1. Introduction

Diet plays an important role in disease prevention and particularly in cases where disease is caused by insufficient, excessive or unbalanced nourishment [1,2]. Epidemiological studies have shown that a plant-based diet can reduce the risk of many chronic diseases [1,3]. Plant foods often contain compounds, known as anti-nutrients, which can potentially interfere with the absorption and metabolism of some nutrients. Saponins for instance can affect nutrient absorption by inhibiting metabolic and digestive enzymes, and by binding with nutrients. On the other hand, studies have demonstrated that saponins may also have various biological and physiological effects when consumed on a regular basis and may be beneficial for the prevention of diseases, such as cancer and coronary diseases [4]. Frequent legume consumption (four or more times weekly compared with less than once a week) has been associated with a lower risk of coronary heart disease (CHD) and cardiovascular disease (CVD) by 22% and 11% respectively [5]. Legume seeds contain a diverse range of bioactive compounds that vary considerably in their biochemistry. These can include protease inhibitors, a-amylases, lectins, glycosides, tannins, saponins and alkaloids [6].

Soya beans appear to be the major source of dietary saponins. The amount of soyasaponins found in soya is typically between 2.4 g kg^−1^ and 6.5 g kg^−1^ [7]. The soyasaponin content depends on the variety, environment, location, and degree of maturity of the soya bean [8]. Soyasaponins are mainly classified into group A and group B soyasaponins. Group A soyasaponins are glycosylated at the C-3 and C-22 position of soyasapogenol A, whereas group B soyasaponins, are glycosylated at the C-3 position of soyasapogenol B [9].

Soyasaponins are believed to be beneficial for human health. Several studies have shown that soyasaponins have anti-carcinogenic, hepatoprotective and antiviral activities, and soyasapogenols exhibit antigenotoxic, hepatoprotective and cytotoxic activities [10,11,12,13]. Our previous work, based on a human intervention study, has demonstrated a significant effect in lowering plasma cholesterol levels following a high protein soy diet for 14 days [14]. Therefore, these molecules represent potential candidates for the development of functional foods and nutraceuticals for the prevention and maintenance of chronic diseases. Since consumers struggle to meet dietary recommendations and to eat a balanced and healthy diet, the food industry is continuously looking to the development of functional foods to make a valuable contribution to people’s diets. Functional foods beneficially affect one or more target functions of the body, beyond adequate nutritional effects. It is pivotal to elucidate the metabolism and bioavailability of a molecule in order to understand its bioactivity and to properly design functional food ingredients.

The presence of soyasaponins and their metabolites in systemic circulation and any related physiological effects may depend on their metabolism by intestinal bacteria. Hu et al. [15] demonstrated that soyasaponin I is poorly absorbed by human intestinal cells and appeared to be metabolised to soyasapogenol B by human intestinal microorganisms and excreted in the faeces. Furthermore, in vivo studies using a rat model suggested that soyasapogenols are much better absorbed than corresponding soyasaponins [16]. The scientific understanding on their bioavailability and biological effects in humans is still not fully elucidated. The aims of the present work are to assess the bioavailability of dietary soyasaponins in humans using a randomised crossover human intervention, and to establish the concentrations and forms of the main metabolites following the consumption of soya-rich diets. Findings from this study provide further insights for the use of soya beans as a functional ingredient in food formulation.

## 2. Materials and Methods

### 2.1. Materials

HPLC grade methanol and analytical grade ethanol were purchased from Fisher Scientific (Leicestershire, UK). LC-MS methanol was purchased from Sigma Aldrich (Dorset, UK) and the Soyasaponin I and Soyasapogenol B standards from Phytolab GmbH & Co. KG (Vestenbergsgreuth, Germany).

### 2.2. Human Dietary Intervention Study

**Figure 1:**
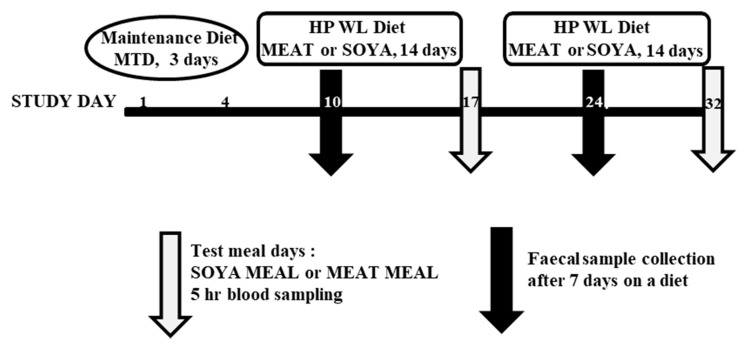
Detailed diagram of the study protocol. After a maintenance diet (MTD) (days 1–3), the order of treatment was randomised using a within-subject, crossover design, whereby 50% of the subjects began with the meat high-protein weight loss (MEAT HP WL) diet (days 4–17) and were then switched to the vegetarian high-proteins weight loss (SOYA HP WL) diet (days 18–31). The other 50% began with the vegetarian high-proteins weight loss (SOYA HP WL) diet (days 4–17) and were then switched to the meat high-protein weight loss (MEAT HP WL) diet (days 18–31). At the end of each WL diet, corresponding to days 18 and 32, volunteers were provided with a test meal (SOYA MEAL or MEAT MEAL) and blood samples were collected at specified intervals within a 5 h stay at the HNU. After seven days on each diet, on day 10 and 24 faecal samples were collected.

Subjects: The study was approved by the North of Scotland Research Ethics Service (NOSTRES) and is registered on ClinicalTrials.gov (http://clinicaltrials.gov/show/NCT02080325). The full study details have been previously described [14]. Briefly, the study protocol was a within-subject crossover design and lasted 31 days as described in the diagram shown in Figure 1. Ten overweight/obese males (body mass index (BMI) ≥ 27 kg/m^2^) were recruited. At days 1–3, the volunteers were asked to consume a maintenance diet (MTD) which contained normal protein; between days 4–17 and 18–31 the volunteers were randomised to either a high protein (HP) weight loss (WL) diet where the protein was comprised predominantly of meat (MEAT HP WL) or a HP, WL diet where the protein was comprised predominantly of soya (SOYA HP WL). Subjects attended the Human Nutrition Unit (HNU) of the Rowett Institute for a breakfast test meal at the end of each dietary period, corresponding to study days 18 and 32. Sample collection: Subjects attended the HNU for a test meal with blood sample collection occurring hourly for five hours. Blood samples were collected using a 20-gauge Venflon^TM^ Intravenous cannula directly into heparinised tubes at the 0, 1, 2, 3, 4 and 5 h time points. The samples were centrifuged (1500× *g*, 15 min; 4 °C) within 45 min to separate the plasma. The harvested plasma was aliquoted and stored at −80 °C until analysis. Only after the deconjugation of volunteers’ plasma with sulfatase and glucuronidase the presence of SSI and SSB in these samples was confirmed. Faecal samples were collected on day 7 of each diet (SOYA and MEAT HP WL) using the commode specimen collection system (Fisher Scientific, Loughborough, UK). Formulation and preparation of the diets: MTD (days 1–3) consisted of 15% protein, 30% fat and 55% carbohydrate fed to 1.5× measured resting metabolic rate (RMR). The SOYA HP WL or MEAT HP WL diet was fed to 100% RMR on a five-day rotation menu, fed as three meals per day containing 30% protein, 30% fat and 40% carbohydrate. MEAT HP WL was based on chicken and beef meat and the SOYA HP WL was based on soya protein or soya-textured vegetable protein. The main soya-containing foods used in the study diet were sourced from the UK market and were identified and analysed for their soyasaponin content. These were soya milk (ALPRO™, Alpro, Ghent, Belgium), margarine (PURE™, Kerry Foods, Warrington, UK), sausages (GRANOSE™, Leeds, UK), powdered isolate (Pulsin Ltd., Gloucester, UK), meat-style slices (Redwood Wholefood Company^®^, Corby, UK), and soya flour (Community Foods Ltd., London, UK).

### 2.3. Extraction and Analysis of Saponins (Soyasaponin I) from Soya Food Products

For the extraction of SSI from soya food products, a previously described method [9] was used with slight modifications. Dried and milled sample (1 g) was extracted with ethanol (70% *v/v*; 10 mL) and stirred for 3 h at room temperature. The mixture was then centrifuged (3220× *g*; 10 min; 4 °C) and the supernatant concentrated to a volume of 3 mL using a rotary evaporator (Büchi R-114, Marshall Scientific, Hampton, NH, USA) at a temperature below 30 °C and pressure 60 mbar. The residue was purified on a Strata C18-E cartridge (6 mL; 1 g from Phenomenex, Torrance USA). This cartridge was activated twice with analytical grade methanol (2 mL) and conditioned twice with water (2 mL × 2). The residue was applied at a very slow flow rate and the polar impurities eluted with water (2 mL × 2). Finally, the product was eluted with methanol (150 mL) and the solvent was removed with the rotary evaporator. The product was dissolved in HPLC methanol (1 mL) and centrifuged (3220× *g*; 10 min; 4 °C). The supernatant was directly injected onto the HPLC. HPLC separation was performed on an Agilent 1290 HPLC system (Agilent Technologies, Wokingham, UK) with a UV detector using a Kinetex C18 column (4.60 × 250 mm, 5 µm particle size; Phenomenex, Torrance USA). The mobile phases used were acetic acid (0.25% *v/v*) in water (solvent A) and acetic acid (0.25% *v/v*) in methanol (solvent B). Gradient elution was performed as follows: 70%–90 % B for 40 min and 90%–60% B for 5 min and then followed by return to the initial conditions (70% B) within 3 min (45–48 min). The injection volume was 20 µL and the flow rate was 1 mL/min. The HPLC chromatograms were recorded at 206 nm.

### 2.4. Extraction and Analysis of Saponins and Sapogenols from Human Biological Samples

#### 2.4.1. Extraction of Soyasaponin I and Soyasapogenol B from Human Faeces

The extraction of SSI and SSB from faecal samples was done according to a published method [15] with slight modifications. The faecal samples were freeze dried and approximately 375 mg of sample was extracted with ethanol (70% *v/v*; 12.5) and stirred for 2 h at room temperature. The extract was centrifuged (3220× *g*; 10 min; 4 °C) and the solvent was removed using a rotary evaporator (Büchi R-114) at a temperature below 30 °C and pressure 60 mbar. The residue was suspended in methanol (20% *v/v*; 5 mL) and purified on a Strata C18-E cartridge (6 mL; 1 g). The cartridge was activated with analytical methanol (5 mL), conditioned methanol (20% *v/v*; 5 mL) and the residue from the evaporation was loaded onto the cartridge at a slow flow rate. The cartridge was washed with water (5 mL), followed by methanol (5% *v/v*; 5 mL) and finally the soyasaponins were eluted with LC-MS methanol (3 mL), and analysed by LC-MS/MS.

#### 2.4.2. Extraction of Soyasaponin I and Soyasapogenol B from Human Plasma

Plasma samples were thawed to 4 °C and transferred (150 µL) to an Eppendorf tube. Sulphatase from Helix Pomatia (S9626-5KU; 0.5 units in citrate phosphate buffer 0.3 mol L; pH 6; 120 µL), and glucuronidase from Helix Pomatia (G7017 136,000 units/mL; 30 µL) from Sigma Aldrich (Dorset, UK), were added. The samples were incubated for 18 h at 37 °C. Methanol (700 µL) containing hydrochloric acid (0.4 mol/L) was added and then the samples were mixed, centrifuged (12,500× *g*; 5 min; 4 °C) and analysed by LC-MS/MS.

#### 2.4.3. In vitro Microbial Transformation of Soyasaponin I by Mixed Human Faecal Microbiota.

SSI (66 µg) in analytical grade methanol was added to sterile amber vials and the solvent was removed under nitrogen. Sterile basal general purpose M2 growth medium (0.5 mL) containing glucose (0.05%; pH 6.5) was then added under a stream of CO_2,_ as described by Miyazaki et al. [17]. The vials were inoculated with microbial faecal slurries (10%; 50 µL) provided by four different healthy human donors consuming a western style diet. Inoculation was conducted in triplicate from each sample donated from each of the human donors. The samples were incubated at 37 °C and were quenched by placing the tubes on ice at 0, 4, 8, 24 and 48 h after incubation. Methanol (LC-MS grade) was added to each vial to a final concentration of 70% and the samples were immediately prepared for LC/MS/MS analysis. Tubes containing the medium plus SSI were incubated for 48 h as a control (n = 3).

#### 2.4.4. LC-MS/MS Analysis of Saponins and Sapogenols from Biological Samples.

LC-MS/MS analysis of SSI and SSB from the human faecal and plasma samples was performed on an Agilent 6490 triple-quadrupole mass spectrometer with an Agilent 1290 Infinity LC, (Agilent Technologies, Stockport, UK). Metabolites were separated on a Kinetex C18 column (250 × 4.6 mm; 5µ) (Phenomenex, Torrance USA). The mobile phases used were acetic acid (0.25% *v/v*) in water (solvent A) and acetic acid (0.25% *v/v*) in methanol (solvent B). Gradient elution was performed as follows: 70%–90% B for 40 min and 90%–60% B for 5 min and then followed by return to the initial conditions (70% B) within 3 min (45–48 min). The flow rate was 1 mL/min which was split post-column before admittance to the mass spectrometer. Ionisation was performed using the Agilent Jet Stream (AJS) source in positive ion mode. Selected source conditions were as follows: Gas Temp 200 °C, Nebulizer 35 psi, Capillary 3000 V, Nozzle 2000 V and Sheath Gas Temp 300 °C. For the first 8 min, data was collected in MS2/SIM mode (set at mass 944.2) for the analysis of Soyasaponins I and then the scan type was changed to multi reaction monitoring (MRM) mode (441.2→423.3) for the analysis of Soyasapogenol B.

### 2.5. Statistical Analysis

All data are expressed as mean ± standard deviation. The effect of treatment over time (on plasma and faecal metabolites) and between metabolites was assessed by two-sided post-hoc t-tests using the volunteers as their own controls. Microsoft ^®^ Excel^®^ for Office 365 (Microsoft Corporation, Redmond, Washington, DC, USA) was used for statistical analyses.

## 3. Results and Discussion

### 3.1. Saponins (Soyasaponin I) Content of Soya-Rich Foods

**Figure 2:**
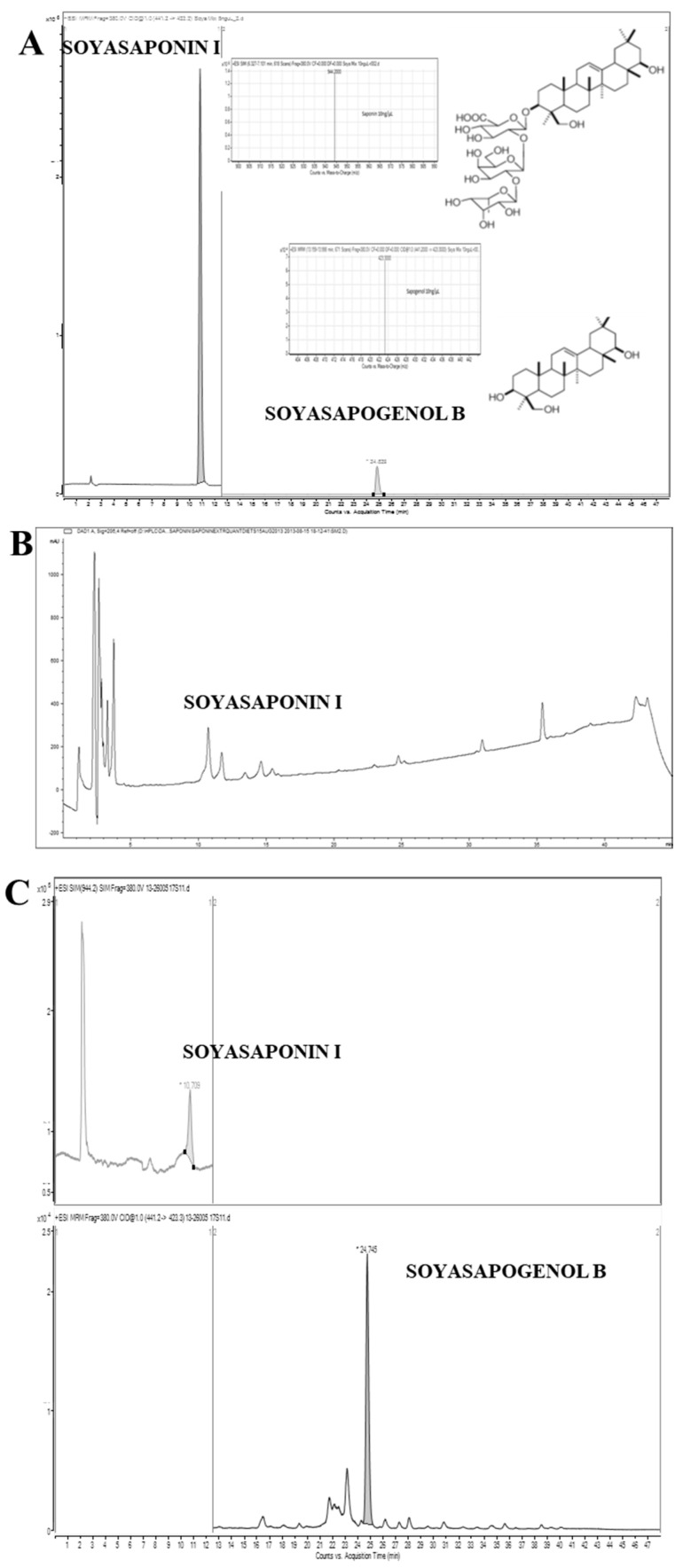
Representative chromatograms for Soyasaponin I and Soyasapogenol B: LC-MS total ion chromatogram for a mixture of SSI and SSB standards showing the extracted mass spectra for each (**A**); HPLC chromatogram (monitored at 206 nm) of soya diet (milk) (**B**); and LC-MS chromatogram of faecal sample obtained from one volunteer after seven days soya diet consumption (**C**).

The main soya products used in the SOYA HP WL diet [14] were analysed for their content of Soyasaponin I (SSI) and the results are presented in Table 1. Representative HPLC chromatograms of soya-rich food products are presented in Figure 2. The soya products were found to be rich in SSI, the richest being the soya protein isolate, followed by soya milk and then soya flour with concentrations of 146.25 ± 10.55, 74.11 ± 4.16, 47.01 ± 5.27 mg in 100 g dry product respectively. Soyasaponin levels in a variety of soya foods have also been determined in previous studies [18,19].

### 3.2. Concentration of Saponins and Sapogenols (SSI and SSB) in Human Faecal Samples

**Figure 3:**
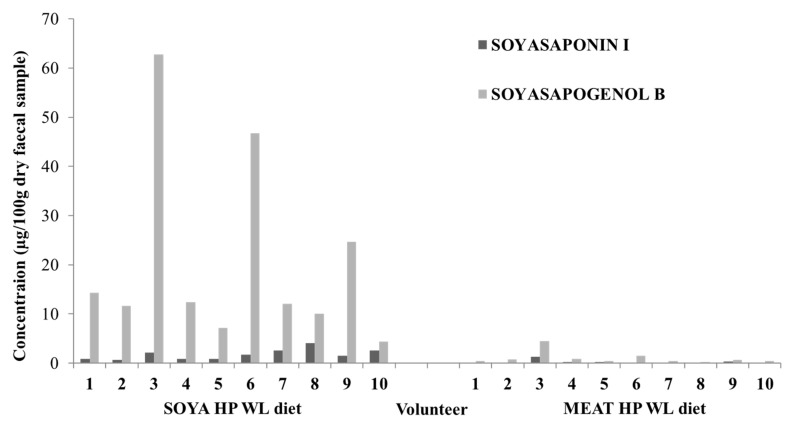
Soyasaponin I (SSI) and soyasapogenol B (SSB) concentrations (mg in 100 g dry sample) in human volunteers’ faecal samples (n = 10) after seven days on the vegetarian high-protein weight loss (SOYA HP WL) diet and meat high-protein weight loss (MEAT HP WL) diet respectively.

Representative LC-MS chromatogram of a volunteer’s faecal sample is presented in Figure 2. The concentration of SSI and SSB in the faecal samples obtained from all volunteers (n = 10) after consumption (7 days) of the SOYA HP WL and MEAT HP WL diets are presented in Figure 3. For the SOYA HP WL diet, the concentration of SSI varied from 0.65 to 4.06 mg per 100 g dry faecal sample (or 0.26 to 1.19 mg in 100 g fresh faecal sample) and the concentration of SSB varied between 4.36 to 62.73 mg in 100 g dry faecal sample (or 1.36 to 17.52 mg in 100 g fresh faecal sample). For the MEAT HP WL diet, the concentration of SS I in human faecal samples varied in the range of 0.04 and 1.31 mg in 100 g dry faecal sample (or 0.01 to 0.38 mg in 100 g fresh faecal sample); and the concentration of SSB varied from 0.26 to 4.52 mg in 100 g dry faecal sample (or 0.08 to 1.32 mg in 100 g fresh faecal sample). Consequently, in the faecal samples both SSB and SSI were present, and SSB was found to be the main metabolite. Similar findings have been previously reported, which also suggest that soyasapogenol seems to be the final metabolic product following anaerobic incubation with human gut microorganisms [20]. The moisture content of the faecal samples after the SOYA HP WL diet was between 58.9% and 72.8% and after the MEAT HP WL diet was between 65.2% and 80.1%. The conversion of SSI to SSB by human faecal microbiota was also tested in vitro.

### 3.3. In Vitro Microbial Transformation of Soyasaponin I by Human Faecal Inoculation

**Figure 4:**
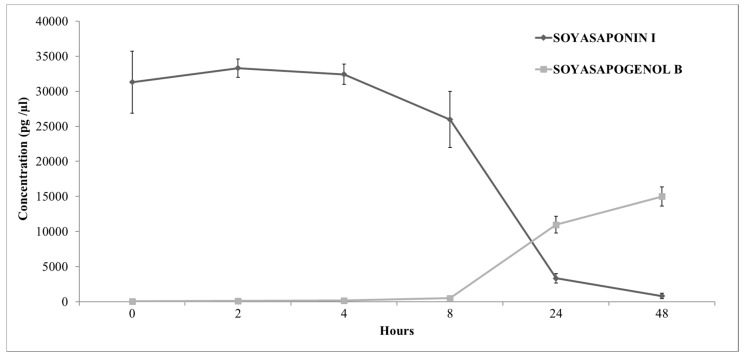
Average Soyasaponin I (SSI) and Soyasapogenol B (SSB) concentrations (µM), for the microbial incubation of SSI with human faecal inoculants (n = 4 donors in triplicate), showing almost complete conversion of SSI to SSB in 48 h.

Previous in vitro research has demonstrated that SSI is converted to SSB by human colonic microbiota [20]. The presence of the parent SSI molecule in human faeces could be explained by the chronic (repetitive) soya consumption of soya-rich foods over the 2-week period. To confirm this hypothesis, and to estimate the length of the time necessary for total conversion of SSI to SSB by the human gut microbiota, in vitro incubation of SSI with human faecal samples (n = 4 donors) was conducted. The results are shown in Figure 4. Incubation for 48 h resulted in almost a total conversion of SSI to SSB (0.98 mol of SSB was recovered from 1 mol of SSI). In the absence of gut microbiota, no SSI degradation into SSB was observed (data not shown). These results agree with published data, which suggests that soyasaponin cannot be detected after a 48 h incubation with human gut enzymes and the main metabolic product is the aglycone form of the precursor molecule (soyasapogenol) [20].

### 3.4. Concentration of Saponins and Sapogenols (SSI and SSB) in Human Plasma Samples

**Figure 5:**
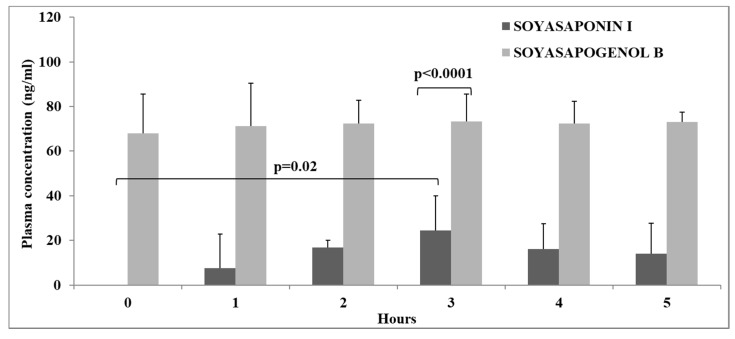
Average Soyasaponin I (SSI) and Soyasapogenol B (SSB) plasma concentrations (ng/mL) throughout the 5 h interval following SOYA MEAL consumption after sulphatase and glucuronidase enzyme treatments. The concentration of SSI at 3 h after consumption of SOYA MEAL was significantly higher in comparison with the baseline (0 h), (*p* = 0.02), and was significantly lower in comparison with the SSB concentration (*p* < 0.0001). Data is presented as mean ± standard deviation for four volunteers.

On test meal days (SOYA MEAL and MEAT MEAL, see Figure 1), blood samples were collected from the human volunteers at regular intervals for up to five hours after test meal consumption. For a sub-sample of volunteers (n = 4) after the SOYA MEAL, the presence of SSI and SSB in plasma was measured for the entire sampling period (0 to 5 h; hourly intervals). As it is common for dietary components to be present in the glucuronidated and/or sulphated form in plasma, the samples were treated with a combined enzyme treatment (sulphatase and glucuronidase). Only after the deconjugation of volunteers’ plasma with sulphatase and glucuronidase, the presence of SSI and SSB in these samples was confirmed (Figure 5). SSB was found in the baseline (0 h) samples and the concentration remained significantly constant (*p* > 0.05) throughout the five-hour sampling period following consumption of the SOYA MEAL. SSI was not detected in the baseline (0 h) samples and the concentration increased to a significant (*p* = 0.02) maximum concentration at three hours following consumption of the SOYA MEAL; however, its absorption systemically was poorer in comparison with SSB. SSB was the major metabolite in human plasma with concentrations (i.e., 73.3 ± 12.1 ng /mL at 3 h postprandial) found to be significantly higher (*p* < 0.0001) than that of SSI (i.e., 24.5 ± 15.5 ng /mL at 3 h postprandial).

These results demonstrate that SSB is the main metabolite present in plasma and that it is found in the conjugated form. The SSB concentration in plasma was constant; there was no significant increase in SSB concentration (after sulphatase or glucuronidase treatment) within the time points analysed (*p* < 0.05).

Taking these results into consideration, plasma samples from all volunteers (n = 10) were analysed for SSI and SSB at baseline (0 h) and at three hours following consumption of the SOYA MEAL. As previously observed, SSB was the main metabolite in plasma and the concentration was stable between baseline and three hours after consumption of the test meals (*p* > 0.05). The average concentration of SSB in the plasma samples before and after the SOYA MEAL was 70.07 ± 14.55 ng /mL at baseline and 74.14 ± 16.04 ng /mL after 3 h. The presence of SSB in fasted plasma indicated that the absorption of the metabolite is most likely to be occurring in the colon and not earlier in the gastrointestinal (GI) tract. The abundance of SSB in human plasma may be related to amount of SSI present in foods (SOYA HP WL diet), however this needs to be further investigated. The absence of SSI in the fasted sample (baseline) and the low detected values in plasma throughout the time course (5 h) indicate very limited absorption early in the GI tract. Therefore, the presence of circulating saponins is predominantly in the conjugated form of sapogenol, which in turn is absorbed following metabolism of the precursor molecule (SSI) by the gut microbiota. Another possible explanation for the low levels of SSI in human plasma could be the complexation of saponins with cholesterol in the gut to form insoluble complexes and/or with bile acids to form mixed micelles [21]. Both proposed mechanisms account for the hypocholesterolemic activity of soyasaponins due to inhibition of intestinal absorption of (endogenous and exogenous) cholesterol and bile acids.

In vitro experiments and animal studies indicate that saponins are poorly absorbed in the intestine. This study has confirmed the bioavailability of dietary soyasaponin aglycones in humans following the consumption of a high soya protein diet for 14 days. Since soyasaponins and their metabolites exert health-promoting properties including the lowering of plasma cholesterol, anti-carcinogenic and hepato-protective effects, understanding their bioavailability and metabolism in humans from soy-rich foods or diets is essential for the design of functional foods. Knowing the extent to which bioactive ingredients reach the systemic circulation from a well-defined food source is the first step for adopting dietary strategies for disease maintenance and prevention. A limitation of the work presented is that the human study was originally designed to measure appetite and weight loss from two different protein sources; however, because of the nature of the study design, the elucidation of systemic bioavailability of soyasapogenol was confirmed. Future work to identify gut bacterial species involved in the process of soyasaponin metabolism will be conducted.

## 4. Conclusions

To date, research suggests that soyasaponins are poorly absorbed early in the GI tract and are metabolised mainly in the colon. There is no evidence to demonstrate that soyasapogenols are systemically bioavailable in the conjugated form. To the best of our knowledge, this is the first report presenting evidence that sapogenols are present in human plasma following microbial metabolism in the colon. Therefore, the proposed mechanism suggests absorption from the colon following metabolism by the gut bacteria. In plasma, SSB is the major metabolite and it is found in the conjugated form.

The present findings are important for further studies to understand the bioactivity of these compounds and the potential development of functional foods containing soya beans. Further work is necessary to validate the suggested anti-inflammatory, hypercholesteraemic and anti-carcinogenic effects of these molecules at nutritionally relevant concentrations and forms in humans. Establishing the bioavailability of bioactive molecules, their form and concentration present in the human gastrointestinal tract represents key information to develop functional ingredients for disease prevention and maintenance.

## Figures and Tables

**Table 1:** Soyasaponin I content in soya rich foods from SOYA HP WL diet.

Soya Product	Soyasaponin I ^a^
Flour	47.01 ± 5.27
Milk	74.11 ± 4.16
Meat	38.97 ± 1.43
Protein Isolate	146.25 ± 10.55
Sausage Spread	26.47 ± 3.73 n/d

^a^ Concentrations are presented as mean ± standard deviation (n = 3) in mg/100 g dry weight, n/d = not detected or below limit of detection.
